# Crops for Carbon Farming

**DOI:** 10.3389/fpls.2021.636709

**Published:** 2021-06-04

**Authors:** Christer Jansson, Celia Faiola, Astrid Wingler, Xin-Guang Zhu, Alexandra Kravchenko, Marie-Anne de Graaff, Aaron J. Ogden, Pubudu P. Handakumbura, Christiane Werner, Diane M. Beckles

**Affiliations:** ^1^Pacific Northwest National Laboratory, Richland, WA, United States; ^2^Department of Ecology and Evolutionary Biology, University of California, Irvine, Irvine, CA, United States; ^3^School of Biological, Earth & Environmental Sciences and Environmental Research Institute, University College Cork, Cork, Ireland; ^4^National Key Laboratory for Plant Molecular Genetics, Center of Excellence for Molecular Plant Sciences, Chinese Academy of Sciences, Shanghai, China; ^5^Department of Plant, Soil, and Microbial Sciences, Michigan State University, East Lansing, MI, United States; ^6^Department of Biological Sciences, Boise State University, Boise, ID, United States; ^7^Ecosystem Physiology, University Freiburg, Freiburg, Germany; ^8^Department of Plant Sciences, University of California, Davis, Davis, CA, United States

**Keywords:** carbon budget, carbon farming, plant-microbe interactions, rhizosphere, rhizosphere microbiome, PGPB (plant growth-promoting bacteria), sustainable agriculture

## Abstract

Agricultural cropping systems and pasture comprise one third of the world’s arable land and have the potential to draw down a considerable amount of atmospheric CO_2_ for storage as soil organic carbon (SOC) and improving the soil carbon budget. An improved soil carbon budget serves the dual purpose of promoting soil health, which supports crop productivity, and constituting a pool from which carbon can be converted to recalcitrant forms for long-term storage as a mitigation measure for global warming. In this perspective, we propose the design of crop ideotypes with the dual functionality of being highly productive for the purposes of food, feed, and fuel, while at the same time being able to facilitate higher contribution to soil carbon and improve the below ground ecology. We advocate a holistic approach of the integrated plant-microbe-soil system and suggest that significant improvements in soil carbon storage can be achieved by a three-pronged approach: (1) design plants with an increased root strength to further allocation of carbon belowground; (2) balance the increase in belowground carbon allocation with increased source strength for enhanced photosynthesis and biomass accumulation; and (3) design soil microbial consortia for increased rhizosphere sink strength and plant growth-promoting (PGP) properties.

## Main

We suggest that significant investments in time and resources should be devoted to developing annual crops for carbon farming. These crops will allocate an increasing amount of carbon to the reproductive sinks, and to the below ground stores for the dual purpose of mitigating global warming due to rising atmospheric CO_2_ levels and improving soil health for increased crop productivity. From a carbon mass balance perspective, this boils down to increasing soil carbon inputs and storage and decreasing outputs, to attain a net increase in soil carbon storage. We posit that this can be achieved through a comprehensive understanding of carbon fluxes and source-sink interactions in an integrated plant-microbe-soil system. Our rationale for focusing on annual crops are four-fold: (1) according to the U.S. Geological Survey, there is a total of 18.6 million km^2^ (4.6 billion acres) of cropland globally (U.S. Geological Survey (USGS), 2019), nearly 80% of which is dedicated to annual agriculture, i.e., cereals, legumes, and oilseed crops (Glover et al., 2007); (2) as opposed to perennials, which require a time commitment of several years, annual crops are more amenable to implementation and deployment of novel and specifically designed crop varieties; (3) perennial plants already invest a substantial portion of their photosynthate in root biomass, and it can be expected that the impact on improved soil carbon status from re-designing perennials will be of less significance compared to efforts on annual crops; and (4) in many cases, annual cropping systems have resulted in a soil carbon debt and, therefore, may be particularly receptive to efforts that aim at improving the soil carbon budget (Griscom et al., 2017; Houghton and Nassikas, 2017; Sanderman et al., 2017).

**FIGURE 1:**
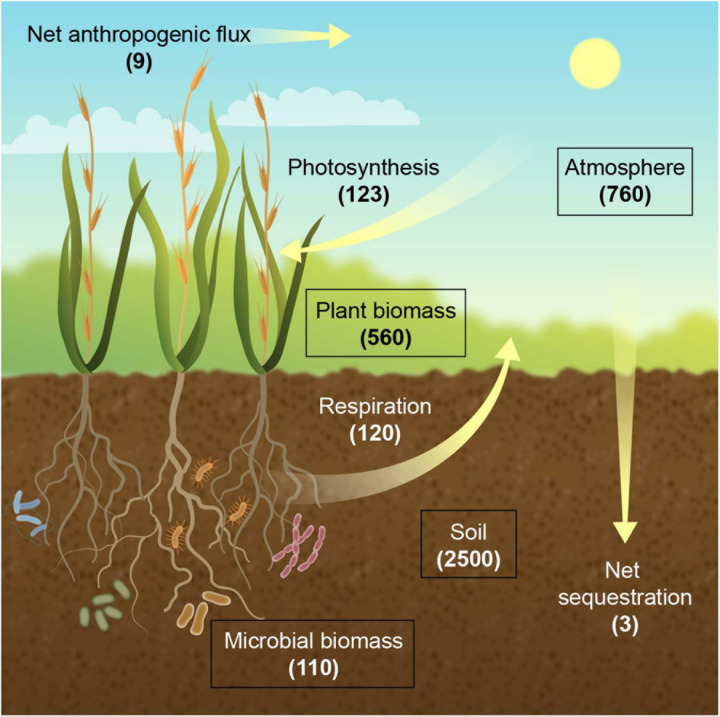
The terrestrial carbon (C) cycle. Carbon stocks (boxes) are shown as gigatons (GT), and fluxes (arrows) are shown in GT per year. Respiration refers to accumulated plant and microbial respiration.

The soil carbon pool with 2,500 gigatons (GT; 1 GT = 1 billion metric tons) in the top 3 m is 3.3 times the size of the atmospheric pool of 760 GT and includes 1,550 GT of soil organic carbon (SOC) and 950 GT of soil inorganic carbon (Lal, 2004a, 2008; Jansson et al., 2010; Figure 1). The SOC pool in the first 1 m and the top 20 cm of the soil profile is 1,500 and 615 GT, respectively (Sanderman et al., 2010; Guo et al., 2016). The primary carbon exchange between the atmosphere and the terrestrial ecosystem is the incorporation of CO_2_ at 123 GT year^–1^ into plant biomass through photosynthesis, of which 3 GT year^–1^ emanates from anthropogenic activities (DOE, 2008)11, and the release of CO_2_ from previously fixed carbon through plant and microbial respiration at 60 and 60 GT year^–1^, respectively (Jansson et al., 2010; Abdullahi et al., 2018). Currently, terrestrial ecosystems are a net carbon sink of 3 GT year^–1^, thereby roughly buffering one third of the annual increase of atmospheric CO_2_ concentration from greenhous gas (GHG) emissions (Le Quere et al., 2018). Consequently, a large fraction of the CO_2_ that is captured as photosynthate is rapidly returned to the atmosphere, and only a minor fraction enters the stable pool of soil carbon. Thus, manipulation of the soil carbon budget, if only by a few percent, represents significant potential for climate change mitigation (Paustian et al., 2016).

Agroecosystems constitute more than one third of the world’s arable land and they contribute 10–14% of global anthropogenic greenhouse gas emissions, primarily from enteric fermentation (methane), application of synthetic fertilizers (nitrous oxide), and tillage (CO_2_) (Jantke et al., 2020). However, agricultural ecosystems also have the potential to store a vast amount of soil carbon (Sanderman and Baldock, 2010; Abdullahi et al., 2018), up to 1 GT year^–1^, which would offset around 10% of the annual GHG emissions of 8–10 GT year^–1^. As stated by the Carbon Cycle Institute, “*Agriculture is the ONE sector that has the ability to transform from a net emitter of CO_2_ to a net sequesterer of CO_2_ —there is no other human managed realm with this potential”* (The Carbon Cycle Institute, 2020). There are numerous land management practices that can be adopted to increase soil carbon storage in agroecosystems, such as changes in crop rotations, tillage, fertilizer management, and organic amendments (Lal, 2004a). Maybe the most effective means for increasing soil carbon sequestration is through changing land cover, such as converting annual cropland to forest or perennial grasses. One caveat with such land use conversions is that it would have negative consequences for biomass yield from the crops that are displaced. One, virtually untapped, alternative option is to select and design annual crop plants that allocate an increased amount of carbon to belowground biomass and root exudates or rhizodeposits.

Soil organic carbon (as a proxy for soil organic matter) plays two roles as we tackle the challenge of achieving sustainable agroecosystems in the coming decades; by increasing crop productivity and by sequestering atmospheric carbon. SOC promotes crop productivity by improving nutrient retention and water holding capacity, by facilitating efficient drainage and aeration, by minimizing loss of topsoil via erosion, and by providing substrates for the soil microbiomes (Lal, 2004a; Sanderman and Baldock, 2010; Berazneva et al., 2019). SOC can be sequestered in persistent pools, e.g., by conversion to biochar or through organo-mineral and organo-metal interactions, with a residence time from decades to thousands of years to millennia (Jansson et al., 2010; Sanderman et al., 2010; Abdullahi et al., 2018). SOC can also be transformed to soil inorganic compounds such as calcium and magnesium carbonates for long-term storage (Guo et al., 2016). The rational design, development, and deployment of crops tailored for carbon farming will depend in part on our ability to model metabolic fluxes of carbon and nitrogen, understand their control, and subsequently apply this insight to reconfigure source–sink interactions and carbon allocation pathways in integrated plant–microbe–soil systems through genome engineering and editing. There is plenty of ongoing efforts in this space to draw from, such as the development of metabolic flux models (Sweetlove and Ratcliffe, 2011; Grafahrend-Belau et al., 2013; Lakshmanan et al., 2016; Töpfer et al., 2020) and genome-scale metabolic networks (Matthews and Marshall-Colon, 2021).

## Where Does the Carbon Go? – A Mass Balance Account

**FIGURE 2:**
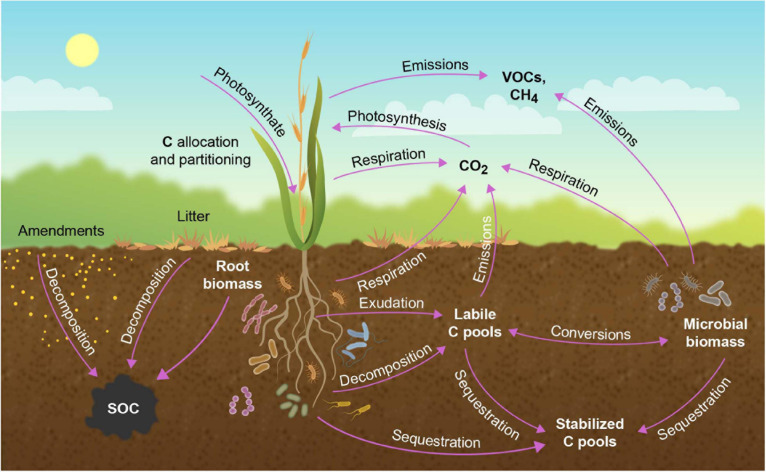
Transfer of atmospheric CO_2_ into biotic and pedologic carbon (C) pools the plant ecosystem. Carbon enters the soil as root exudates or via decomposition of root or aboveground biomass. In the soil, C exists in root or microbial biomass, as bioavailable labile organic C, or as more recalcitrant C. Carbon exits the soil as direct emissions, or via root or microbial respiration, with microbial-mediated soil respiration being the major source of CO_2_ from terrestrial ecosystems. Carbon is also lost from the ecosystem as volatile organic compounds (VOCs) and methane (CH_4_). Modified from Jansson et al. (2018).

A representation of carbon fluxes in a terrestrial ecosystem is depicted in Figure 2. The mass balance for carbon (and any material) in the ecosystem can be accounted for by the equation

(1) I⁢n⁢p⁢u⁢t-O⁢u⁢t⁢p⁢u⁢t=S⁢t⁢o⁢r⁢a⁢g⁢e

For carbon, Eq (1) can be summarized as follows:

*Inputs:* Photosynthesis; Soil amendments

*Outputs:* Plant and microbial respiration; Emissions of volatile organic compounds (VOCs)

*Storage:* Plant and microbial biomass; Soil carbon as SOC and *SIC* pools

The soil carbon content is governed by the balance between photosynthetic inputs via liter and root exudation and amendments such as manure and compost, and outputs through heterotrophic respiration from roots and microbes and via VOC emissions.

Carbon allocation is a crucial physiological process where assimilated atmospheric CO_2_ (photosynthate) is shifted between respiration and biomass production, transient and enduring tissues, and aboveground and belowground components. Functional, or optimal equilibrium theory holds that plants allocate resources among organs to optimize whole-plant fitness (Thornley, 1972; Bloom et al., 1985; Poorter and Nagel, 2000). Thus, allocation of recent photosynthate between aboveground and belowground biomass for a given plant will shift in response to environmental variables such as soil moisture, light, and nutrient availability. Evidence from field studies indicates that plants allocate relatively more carbon to shoots under light limitation and to roots under water and/or nutrient limitation.

Plants allocate considerable amounts (20–30%) of recent photosynthate to their belowground biomass. About 50% of the translocated carbon is used for root growth, while a substantial fraction of this carbon (up to 30%) is further released to the rhizosphere, either as direct root deposition through exudation, sloughed root cap cells, or via mycorrhiza, or is lost through respiration (Lorenz et al., 2008; Turner et al., 2013; Kaiser et al., 2015). Both root exudation and transfer to mycorrhizal fungi occur rapidly after photosynthesis, ranging from a few hours in grasses to a few days in trees (Kaiser et al., 2015). Root exudation stimulates microbial decomposition of SOM, which in turn improves nutrient availability along the rhizosphere. Carbon transfer to mycorrhizal fungi benefits the plant through direct nutrient transfer from the fungal hyphal network. In both cases, the plant’s investment in belowground carbon allocation is rewarded with increased nutrient availability, in particular nitrogen and phosphorus, as well as enhanced tolerance to abiotic stress such as drought, heat, and salinity (Kaiser et al., 2015; Begum et al., 2019).

A substantial portion of the photosynthate can be released to the atmosphere in the form of VOCs, with isoprenoids representing the dominant compound class (Guenther et al., 2012). The amount of carbon re-emitted as isoprene (with this single compound representing about half of all isoprenoid emissions) generally comprises 1–3% of NPP but can significantly increase up to 50% given unfavorable environmental conditions, particularly when photosynthetic carbon uptake is low (Loreto and Sharkey, 1993; Sharkey and Loreto, 1993; Harley et al., 1999). Thus, a larger fraction of fixed carbon is re-emitted under conditions of moderate plant stress while total emissions decrease under extreme stress (Holopainen and Gershenzon, 2010; Niinemets, 2010; Niinemets et al., 2013). These VOCs are highly reactive and readily participate in atmospheric oxidation chemistry (Atkinson and Arey, 1998, 2003). Oxidation products of these reactions undergo gas-particle partitioning and contribute to the formation of atmospheric particulate matter, called secondary organic aerosol (SOA; Hallquist et al., 2009; Mohr et al., 2019). Atmospheric aerosols influence radiative transfer through the atmosphere directly by scattering and absorbing incoming solar radiation and indirectly by contributing to cloud formation processes (Kerminen et al., 2005; Spracklen et al., 2008; Riipinen et al., 2012). The type of volatiles emitted from different plants under healthy and stressed conditions can vary greatly with substantial impacts on SOA formation (Mentel et al., 2013; Joutsensaari et al., 2015; Yli-Pirilä et al., 2016; Zhao et al., 2017; Faiola et al., 2018, 2019). This influences the quantity and characteristics of the light available for plant use, including the ratio of diffuse to direct light with subsequent impacts on NPP (Rap et al., 2018). These relationships are illustrated schematically in Figure 2 and highlight potential feedback mechanisms between the soil-plant-atmosphere system mediated through VOC emissions and aerosol production. Significant impact of VOC-mediated secondary aerosol formation on atmospheric processes and human health have been observed e.g., from the conversion of crop fields to isoprene emitting bio-fuel plants (e.g., poplar) (Ashworth et al., 2013). Accounting for these feedbacks in making decisions about carbon farming will be particularly important under future climate scenarios with increased drought stress, which could increase the overall proportion of carbon re-emitted in the form of VOCs. We caution, that most VOC studies to date have been performed in perennials, and the relevance of aspects discussed above to annual crops such as sorghum, corn, wheat, and soybean, needs to be addressed through large-scale assessments.

## Increase Root and Rhizosphere Sink Strength

**FIGURE 3:**
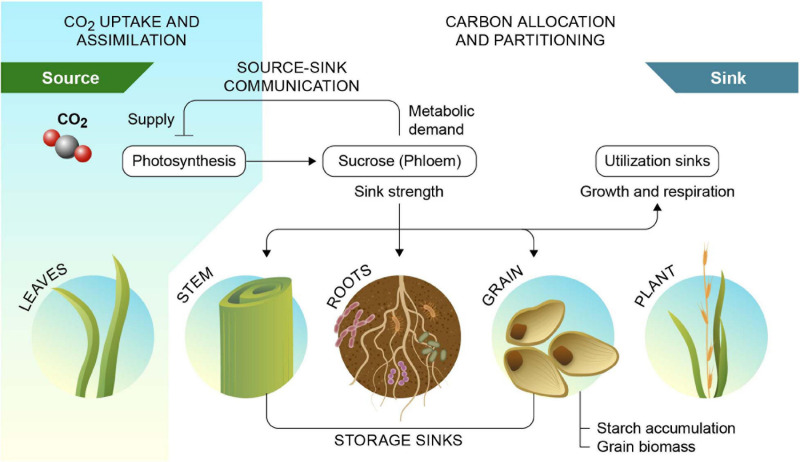
Source–sink interactions of photosynthate production and utilization. Source-sink interactions link carbon sources such as mature leaves to sinks such as roots and seeds and mediates feedback inhibition of photosynthesis via perceived sink demand. Sink strength of the rhizosphere is contributed by the root biomass and associated microbial communities, including arbuscular mycorrhiza. Reprinted from Fan et al. (2008).

Soil carbon stocks can be augmented by increasing the rate of carbon additions to the soil, by increasing retention of new carbon deposited in soil, or by reducing the rate of decomposition of SOC already present in the soil. A potential path to increased soil carbon stocks is the employment or development of crop cultivars that input a greater quantity of carbon into the soil through their roots. Just like sink strength of developing grains is a key determinant of grain yield, utilization of carbon for root growth determines root sink strength and belowground accumulation as SOC (Figure 3). This may be achieved by a greater root biomass, or by a greater surface area of roots that actively release carbon into soil. For example, switchgrass cultivars with a greater proportion of fine roots, and a relatively large specific root length enhanced soil carbon input in bioenergy cropping systems (Adkins et al., 2016). However, for long-term sequestration of this carbon, it is essential that it is retained in soil, either through associations with soil minerals, via conversion to carbonate minerals or recalcitrant organic carbon like charcoal, or via reduction of microbial respiration, i.e., an increase in microbial carbon-use efficiency. Growing deeper root systems presents another pathway by which soil carbon input and retention may be enhanced. Carbon deposited at depth may have a greater mean residence time, because decomposition rates are slower in deeper soil profiles compared to surface horizons, promoting long-term soil carbon storage. Additionally, deeper roots can to some extend buffer the impacts of droughts, thus further increasing carbon uptake. If developed, such plants could be deployed rapidly, and at scale, due to continuous genetic turnover and active land management in agricultural croplands. Improving plants to increase soil carbon sequestration represents an untapped and economic net carbon sink with significant economic potential (Paustian et al., 2019a). A rationale for this concept was presented by Kell (2012). Further, under scenarios of elevated atmospheric CO_2_ levels due to climate change, C_3_ crops that are typically CO_2_ limited may instead face nitrogen limitation, making a larger root biomass even more advantageous.

In an analysis to support a new program launched by the US Department of Energy, Paustian et al. (2016) performed a “bounding analysis” to estimate what level of soil carbon increase and total greenhouse gas mitigation (including N_2_O emissions) might be possible based on specifying feasible increases in total root mass and changing root depth distributions toward those found in perennial grasses. They estimated that widespread adoption of annual crop phenotypes designed to have deeper and larger root systems could yield soil carbon stock increases of 0.5 GT CO_2_ ha^–1^ year^–1^ on current US cropland (Paustian et al., 2016, 2019a).

While it is well known that root systems play a major role in SOC supply and storage, exactly which root characteristics are important for maximizing SOC gains and for ensuring long-term carbon storage is not obvious. High root-derived carbon inputs are a necessary-but-not-sufficient prerequisite for soil carbon gains. For example, greater root biomass and root exudation, stimulated by higher CO_2_ levels, do not necessarily translate into high soil carbon gains, a phenomenon explained by enhancement of microbial activity and enhanced priming of old soil organic matter (Hungate et al., 1997; Pendall et al., 2004; Phillips et al., 2012) (yet see Jastrow et al., 2005). A nine-year-old field trial comparing two perennial herbaceous systems, monoculture switchgrass and highly biodiverse native succession vegetation, demonstrated that, even though switchgrass root biomass more than 10-fold exceeded that of native vegetation, improvements in SOC levels under switchgrass were noticeably lower (Kravchenko et al., 2019). These examples suggest that it is not at all certain that breeding for higher root biomass is the answer to faster and more effective SOC storage. Among the plant characteristics that should be considered as such that might lead to SOC gains are: (1) physical characteristics of the root system architecture—not only the total root biomass, but also the root morphology auspicious for soil structure formation (Bardgett et al., 2014; Postma et al., 2017; Kravchenko et al., 2019; Voges et al., 2019); (2) quantities of carbon entering the soil in a form of rhizodeposits during root growth and live functioning (Kuzyakov and Domanski, 2000; Bengtson et al., 2012; Mwafulirwa et al., 2016); (3) chemical composition of root tissues and exudates (Keiluweit et al., 2015; Naveed et al., 2017); and (4) development of a rhizosphere microbiome able to convert root carbon inputs into protected SOC with greater efficiency (Lange et al., 2015).

Yet, possibly none of these factors matters by themselves, but, instead, a favorable combination of all is needed to generate SOC gains (Yang et al., 2019). As alluded to above, how much carbon stays in soil depends on how much is put in and how much remains protected. Recent studies have revealed that low molecular weight root exudates are particularly important for SOC formation and retention. This is because they promote microbial residue formation, and microbially derived compounds make up a large proportion of stable SOC (Cotrufo et al., 2013; Kallenbach et al., 2016). However, many questions remain regarding the interactions among root exudation, the microbial community structure, its physiology, and ultimately impacts on the ratio of carbon turned into microbial biomass versus the amount of carbon released from soil as CO_2_. The ideal scenario might be large amounts of high-quality root-derived carbon inputs accompanied by formation of the pore architecture favorable for protecting the new carbon from further decomposition (Kravchenko et al., 2019), i.e., by greater heterogeneity of the pore space (De Deyn et al., 2011).

It also would need to be considered that, even within the same species/genotype, the specific contributions of individual plants can depend on their age, their growth conditions in terms of nutrient and water supply and presence of stresses (Uren, 2007; Helliwell et al., 2019), and the competition with neighboring plants of the same or different species/genotypes (Fan et al., 2008). In perennial grassland vegetation complementarity of high plant diversity has been shown to be highly beneficial to soil carbon storage; high diversity treatments performed significantly better than any monocultures (Yang et al., 2019) and biodiversity in terms of greater number of plant species appeared to be more important than the plant biomass inputs (Steinbeiss et al., 2008). Those are facets that will need to be considered in deciding where to direct the engineering efforts in modifying root systems of annual plants to increase their ability to protect SOC.

In addition to plant diversity and root morphology, altering the quantity and composition of root exudates to increase SOC is an attractive focus area for two reasons. First, predictably controlling total root exudate production can directly impact the source-sink relationship between root and aerial plant organs. Second, the composition of root exudates impacts rhizosphere microbial community succession, and therefore allows breeders a way to potentially steer composition of crop rhizobiomes in favor of microbes that are better carbon sinks, improve soil characteristics, or increase longevity of SOC. The role of AM fungi as carbon sinks for plants is well documented (Churland and Grayston, 2014; Gorzelak et al., 2015; Kaiser et al., 2015), and there is evidence that bacteria and other members of the rhizospheric microbiome provide similar functions (Trivedi et al., 2013; Kallenbach et al., 2016). For example, it was recently shown that plant-derived coumarins in root exudates limit growth of particular bacterial taxa in the rhizobiome (Voges et al., 2019). Thus, controlling total root exudate production as a strategy for increasing root sink strength, as well as exudate composition as a strategy to influence rhizosphere community dynamics should be explored as an option for allocating more carbon to the soil for long-term storage. Here, tipping the compositional balance of the rhizosphere microbiome in favor of AMF may provide the necessary benefits for yield as well as for soli carbon storage (Berger, 2019; Zhang et al., 2019).

**FIGURE 4:**
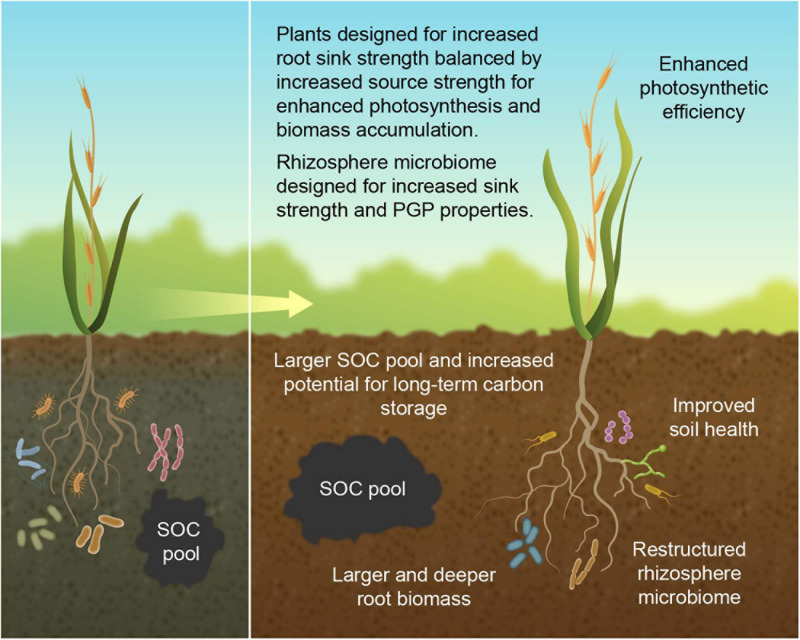
Rationale for designing an integrated plant-microbe-soil system with the dual goal of improving the soil carbon budget while maintaining crop yield, showing current crop **(left panel)** and desired crop ideotype **(right panel)**. Larger root biomass confers increased sink strength that funnels more carbon to the soil, and deeper roots increase the likelihood for long-term soil carbon storage. Custom-made rhizosphere microbiomes are designed to further augment the demand for belowground carbon, thereby increasing the rhizosphere sink strength, while also providing PGP properties. To complement promotion of plant productivity from improved soil health, PGP microbes, and enhanced photosynthesis by increased sink demand, plants are also designed for increased source strength to further enhance photosynthesis and biomass accumulation.

It is well documented that the plant microbiome exerts a plethora of plant growth-promoting (PGP) effects that benefit their host plants, such as conferring enhanced abiotic and biotic stress tolerance and improved nutrient acquisition, including N-fixation (Ahkami et al., 2017). Thus, tailoring a rhizosphere microbiome to combine an increase in sink strength with enhanced PGP properties should go a long way toward achieving the dual goal of improving the soil carbon budget and boost productivity (Figure 4). We envision that, while designing microbiomes with assigned functional properties will leverage the enormous existing soil microbial diversity, it will also encompass the possibility of employing genome-engineering/editing to construct highly specific synthetic microbial communities (SynComs) that are obligate symbionts to the plant host, so as to provide a biocontainment strategy. The design and utility of SynComs in improving plant traits were recently discussed in de Souza et al. (2020).

## … Without Jeopardizing Yield

Designing crops for carbon farming and improving the soil carbon budget is unlikely to be a viable approach unless farmers see a financial return. Although such returns could take the form of tax credits and cap-and-trade programs, this does not provide a sustainable, long-term solution. While improving soil health by increasing the SOC pool and benefiting from increased PGP effects of synthetic rhizosphere microbiomes will ultimately favor plant productivity, it is unlikely to maintain, let alone increase, crop yield in plants designed for funneling a substantial portion of photosynthate to the soil. Therefore, carbon farming will also need to include measures to enhance photosynthetic energy conversion efficiency and hence biomass production. Several such options have been explored (Ort et al., 2015; Kromdijk et al., 2016; Jansson et al., 2018; South et al., 2019). In particular, an increase in the speed of recovery from photoprotective state resulted in increased photosynthesis and biomass production (Kromdijk et al., 2016). Similarly, engineering photorespiratory bypasses can result in increased CO_2_ levels around Rubisco and decreased photorespiratory lost (South et al., 2019). Modification of leaf biochemical and anatomical features and decreasing the resistance of CO_2_ diffusion to the site of its fixation around Rubisco has the potential to increase source activity without increase water loss through stomata (Flexas et al., 2016). Increasing the speed of adjustment of stomatal conductance or activities of Calvin Benson cycle under fluctuation light can also increase photosynthetic efficiency and crop water use efficiency (Lawson and Blatt, 2014; Taylor and Long, 2017). In addition to these, radical manipulations of photosynthetic machinery, such as engineering CO_2_ concentrating mechanisms (CCMs), such as C_4_ photosynthetic machinery or cyanobacterial carboxysome CCM into C_3_ crops also hold great potential to dramatically increase source activity (Long et al., 2015). The major assumption here is that to have these options useful in increasing crop yield and root biomass, the increased photosynthetic rates will not result in decreased allocation to root tissues. Indeed, when plants of the same functional type are considered, relatively conserved root:shoot ratios are obtained, though plants of different functional types, or grown under different environment, or at different growth stages show drastically different root:shoot ratios (Mokany et al., 2006). We recognize that our knowledge of biochemical pathways in plants are often not sufficiently complete that we can reasonably predict the consequences of changing the expression of targeted genes. Here, open-ended forward genetics approaches through genome editing may be useful as a complement to rational design (Zsögön et al., 2017; Andres et al., 2019; Belcher et al., 2020).

The growth and yield of crops are not solely limited by photosynthesis in mature leaves (source strength) but also by the allocation pattern of photosynthates to other organs, i.e., sink strength. Importantly, if sink strength does not keep up with source strength, this can result in the accumulation of carbohydrates in the leaves and feedback inhibition of photosynthesis (White et al., 2016; Jansson et al., 2018; Figure 3). Since elevated atmospheric CO_2_ concentration increases source more than sink strength, it will become even more important to focus on enhancing sink strength alongside photosynthesis in crop improvement (Chang and Zhu, 2017). Increased yield and plant carbon capture could be achieved by optimizing the regulatory processes that determine sink strength in heterotrophic organs, in combination with overcoming the feedback inhibition of photosynthesis. It is conceivable then, that an increased sink strength in the roots or rhizosphere to draw more carbon to the soil can be combined with maintained, or even enhanced, yield by capitalizing on elevated atmospheric CO_2_ levels and/or by uncoupling photosynthesis from feedback inhibition by sink demand. This is particularly true for C_3_ crops that will directly benefit from increased source strength by rising CO_2_ concentrations.

In considering the scenario depicted above, it is critical to understand that the extent to which a crop plant is source or sink limited depends on its developmental stage and the environment and also varies between species and genotypes. Annuals typically transition from sink to source limitation during development when they switch from vegetative to reproductive growth (Arp, 1991; Burnett et al., 2016), although cereals such as wheat and barley can remain sink limited during reproduction (Serrago et al., 2013). Perennials, in contrast, may evade sink limitation and acclimation to elevated CO_2_ through developmental plasticity (Burnett et al., 2016).

Another important aspect is the diversification of crop rotations and annual coverage. Long-term experiments and recent eddy covariance measurements have shown contrasting results regarding the CO_2_ sink or source function of croplands, which vary between cultivar and management techniques (Poyda et al., 2019). The carbon uptake during the growing season can be compensated by stronger heterotrophic respiration from bare soils after harvest (Schmidt et al., 2012). Inclusion of cover crops (rather than allowing a fallow period during the winter months) can increase the SOC stock of cropland soils and thus be another effective measure to compensate CO_2_ emissions. Thus, cover cropping has been shown to improve the net ecosystem carbon balance by replacing the bare fallow period when carbon is lost by soil respiration, by an additional period of carbon assimilation (Lal, 2004b). A recent meta-analysis has estimated a potential global SOC sequestration of 0.12 Pg C year^–1^, which would compensate for 8% of the direct annual greenhouse gas emission from agriculture (Poeplau and Don, 2015). Intercropping has often been applied to either improve soil nutrition, e.g., with N-fixing plants, or inversely to secure uptake of excess N and reduce soil N leaching, and enhance P and soil microbial communities. Thus, cover crops have been mainly investigated for their capacity to improve soil quality (Hallama et al., 2019). Another untapped possibility to mitigate climate change effects rests upon engineering cover crops with deeper root systems and enhanced soil carbon allocation provides.

## The Need for Modeling Fluxes

Development of comprehensive, highly mechanistic systems models of crop growth and development (Chang and Zhu, 2017) to guide the design new integrated crop-microbe systems or agronomic practices is highly desirable. For example, since roots are heterotrophic organs, a larger root biomass will further increase an already significant root respiration. The ability to accurately predict carbon losses through respiratory fluxes becomes an important tool in crop design. More generally, around 50% of assimilated photosynthate is subsequently lost to respiration (Amthor et al., 2019). Minimizing “non-essential” respiratory activity leading to unnecessary CO_2_ release is unlikely to have been subject to selection pressure during evolution or being considered in crop breeding programs and cutting this large loss could complement and reinforce other efforts in designing crops for enhanced productivity. Such strategies to decrease respiratory cost could include: (1) slow unnecessary protein turnover; (2) replacing, relocating, and/or rescheduling metabolic activities; (3) suppressing futile cycles; (4) improving ion transport efficiency (Amthor et al., 2019). A comprehensive understanding of short- and long-term carbon (^13^C) and nitrogen (^15^N) fluxes within a model cereal could be instructive, displaying the proportion of carbon allocated to the source and sinks (grain and roots), and the percentage released to the soil and the atmosphere. This integrated understanding of carbon movement would be viewed in relation to plant growth, biomass, yield, and plant productivity. This data would serve as a blueprint from which to understand developmental and environmental shifts in carbon allocation, to begin engineering strategies, and to better support predictive models. For example, toward understanding the effects of reduced nitrogen, higher CO_2_, drought, and heat, singly, and in different combinations, on the plant-microbe-environment carbon continuum. Another example to highlight is the need for measuring and predicting fluxes relates to the emissions of N_2_O (the most potent biogenic greenhouse gas on a per mass basis) resulting from increased nitrogen inputs when needed to match elevated atmospheric CO_2_ levels. Models are also required that account for sink stimulation of photosynthesis to assess the extent by which carbon costs incurred as part of interactions or symbioses with the rhizosphere microbiome are compensated for by enhanced photosynthesis and biomass production (Kaschuk et al., 2009). Development of predictive metabolic models requires detailed maps of metabolic fluxes in different organs and across the whole integrated plant-microbe-soil-atmosphere system under different conditions, and how these change under changed environmental or internal conditions. This represents a major area of research for the future plant environmental and molecular physiology. Metabolic flux analysis is widely used to study microbial communities and it can be adapted to derive comprehensive map of photosynthate and compound transfer between different organs in crops and other plants (Wiechert, 2001; Ma et al., 2014).

## Closing Remarks

Carbon farming aims to improve the rate at which CO_2_ is removed from the atmosphere and converted to plant material and soil organic matter. The positive outcome of this ambition is two-fold; improved soil health, which, in turn, promotes crop productivity, and increased potential for long-term carbon storage to mitigate greenhouse gas emissions. In this perspective we argue that crops designed for carbon farming should be endowed with the following attributes: (1) increased belowground carbon allocation for larger and deeper root biomass; (2) interactions with a tailored, synthetic soil microbiome for increased rhizosphere sink strength and enhanced PGP properties that facilitate nutrient acquisition and water-use efficiency; and (3) increased source strength for enhanced photosynthesis and biomass accumulation. This represents an ambitious initiative that entails genome engineering/editing of the integrated plant–microbe–soil system, supported by systems-level multi-omics analysis, and metabolic flux analysis and modeling. It will also be important to engage international breeding programs and cull resources from extensive germplasm collections (Lenaerts et al., 2019; Voss-Fels et al., 2019). This becomes particularly relevant in efforts to integrate and balance traits for soil carbon deposition with biomass yield and stress tolerance and resilience.

We conclude by recognizing that soil carbon storage is not an infinite solution to curtailing greenhouse gas emissions, primarily since soils have an upper limit or saturation level of carbon (Six et al., 2002; Paustian et al., 2019b). We recognize the challenges associated with societal acceptance of genome-edited crops and new agronomic practices in implementing crops for carbon farming. However, carbon farming offers the opportunity in the next coming decades to capitalize on the substantial potential inherent in combining agriculture with the rhizosphere microbiome in promoting soil carbon sequestration. As such, designing crops for carbon farming aligns with the consensus from the Paris climate agreement, stating that economically optimal paths to reach the Paris goal in limiting global warming not only requires cutting emissions of greenhouse gasses but must also include negative emissions technologies, such as stimulating the soil to store more carbon (Economist, 2017).

## Data Availability Statement

The original contributions presented in the study are included in the article/Supplementary Material, further inquiries can be directed to the corresponding author.

## Author Contributions

CJ conceived the work. All authors contributed to the article and approved the submitted version.

## Conflict of Interest

The authors declare that the research was conducted in the absence of any commercial or financial relationships that could be construed as a potential conflict of interest.
